# Association between Healthy Lifestyle (Diet Quality, Physical Activity, Normal Body Weight) and Periodontal Diseases in Korean Adults

**DOI:** 10.3390/ijerph19073871

**Published:** 2022-03-24

**Authors:** Su-Yeon Hwang, Jong-Hwa Jang, Jung-Eun Park

**Affiliations:** 1 Research Institute for Future Medical Science, Chungnam National University Sejong Hospital, Sejong 30099, Korea; hsyen@naver.com; 2 Department of Dental Hygiene, College of Health Science, Dankook University, Cheonan-si 31116, Korea; jhj@dankook.ac.kr

**Keywords:** periodontal disease, healthy eating index, body mass index, physical activity, healthy lifestyle

## Abstract

This study aimed to investigate the association between healthy lifestyle (HLS; i.e., diet quality, physical activity, normal weight) and periodontal diseases in Korean adults. Studying this association may help inform future intervention programs aimed at preventing the development of periodontal diseases. Raw data of the Korea National Health and Nutrition Examination Survey (KNHANES) VII (2016–2018) were used. Data from 12,689 adults aged 19 years and over who had a periodontal examination were analyzed. The associations between HLS and periodontal diseases were analyzed using multivariate logistic regression after adjusting for demographic and health factors as covariates. We found that each of the HLS (diet quality, physical activity, normal body weight) practices was significantly associated with periodontal diseases (OR: 1.32, 95% CI: 1.13–1.55; OR: 1.16, 95% CI: 1.04–1.30; OR: 1.26, 95% CI: 1.14–1.40, respectively). In particular, having poor HLS practices was identified as a risk factor for periodontal diseases (OR: 1.54, 95% CI: 1.10–2.15). HLS was associated with periodontal diseases. Thus, in addition to improving oral hygiene—the primary focus in the past—improving HLS should be emphasized for patients with periodontal diseases.

## 1. Introduction

Periodontal diseases are chronic inflammatory diseases affecting the tissues surrounding the teeth that are highly prevalent worldwide. Periodontal diseases are caused by bacterial biofilms that lead to the loss of adhesion of periodontal tissues [1]. Moreover, periodontal diseases cause resorption of the connective tissue and alveolar bone in the oral cavity, which in turn leads to tooth loss in adults [2].

In 2018, the economic cost of periodontal disease was $154.06 billion in the U.S. and €158.64 billion in Europe [3]. Therefore, prevention and management of periodontal diseases associated with a large economic burden are necessary.

Chronic diseases, including periodontal diseases, are known to affect the life expectancy and morbidity of individuals based on various lifestyle factors, including smoking, physical activity, alcohol consumption, body weight, and diet quality [4,5,6]. Thus, identifying modifiable lifestyle practices is crucial to the effective prevention and management of periodontal diseases. Periodontal disease treatment and management providers play a key role in supporting patient health behaviors and face the challenge of intervention for behavior change [7]. Therefore, intervention and behavioral support are needed to promote changes in the lifestyle factors.

According to previous studies on health behaviors that determine HLS, high diet quality was significantly associated with the number of teeth [8]. A protective diet is recommended as diet-related systemic inflammation is associated with periodontal diseases and tooth loss [9,10]. Moreover, elderly patients with periodontal disease tended to consume inflammation-prone food [11].

Furthermore, adequate physical activity was associated with reduced incidence of periodontal diseases, based on which it has been identified as a protective factor against periodontal diseases [12]. The risk of periodontal illnesses increased among overweight individuals, and there was a significant association between obesity and periodontal diseases [13]. A systematic review of HLS reported that HLS interventions targeting smoking, diabetes mellitus control, physical activity, dietary changes, reduced carbohydrate intake, and weight loss improved periodontal tissues [7].

Some studies have found an association between periodontal diseases and HLS comprising diet quality, physical activity, and weight control [14,15]. One of these studies sheds light on the association between HLS and periodontal diseases in the US adult population; however, the data do not show the specific relationships with each of the HLS practices (diet quality, physical activity, and weight control) [14]. Another study showed that exercise and healthy diet were linked in reducing the incidence of periodontal diseases in Jordanian adults; however, this study did not present data on the association with body weight control [15].

Many risk factors of periodontal diseases overlap and may affect causality [16]. Moreover, interventions to improve diet, physical activity, and healthy weight may be mutually interdependent. For example, interventions aimed at controlling risk factors in patients with type 2 diabetes mellitus and periodontal diseases include a combination of diet therapy, physical activity, and weight loss regimens [7]. Therefore, the aim of this study is to investigate the association of periodontal diseases with HLS and the specific practices of HLS, namely diet quality, physical activity, and body weight control, in the Korean adult population. We hypothesized that the risk of periodontal disease is lower for people with higher HLS practices.

## 2. Materials and Methods

### 2.1. Study Participants

We used data from the Korea National Health and Nutrition Examination Survey (KNHANES) VII (2016–2018), a nationally representative cross-sectional survey. The first- and second-year surveys (2016–2017) were considered as research conducted by the nation for public welfare and thus were exempted from review by the Institutional Review Board at Korea Disease Control and Prevention Agency (KDCA) per the Bioethics and Safety Act. The third-year survey (2018) was reviewed in consideration of the collection of human-derived materials and third-party disclosures of raw data (IRB No. 2018-01-03-P-A).

The KNHANES data are released for public use [17], and the study population is a complex sample selected by proportional allocation and systematic sampling. The KNHANES VII (2016–2018) data used in this study were obtained from surveys conducted on all members (age ≥ 1 year) of 4416 sample households nationwide from January 2016 to December 2018. The latest census data available at the time of sampling design were used as the base sampling frame to obtain a representative sample. To use the latest data reflecting the current characteristics of the population and improve the population inclusion rate, the base sampling frame was complemented with the officially assessed multi-unit house prices. Two-stage stratified cluster sampling was used, where the enumeration districts and households are set as the sampling units in the first and second stages, respectively. The sampling frame was stratified by neighborhood (dong), town (eup), or township (myeon) and type of house (single or multi-unit houses, apartments); the proportion of residential area and rate of education level of head of household were used as implicit stratification criteria. From 192 enumeration districts, nursing homes, prisons, military facilities, and foreigner households were excluded, and a total of 23 sample households were selected through systematic sampling. From the participant data, we used the oral examination data. KNHANES shows indicators on health behaviors (smoking, drinking, physical activity, etc.), screening examination (hypertension, diabetes, oral diseases, etc.), and nutritional status (food and nutrient intake, dietary behavior, etc.). KNHANES data were collected through the self-administered method, interviews, direct measurement, or sample analysis. In this study, oral examination data of KNHANES were used for analysis.

A total of 13,199 adults aged 19 years and over were selected from 16,489 participants. Of them, 12,689 participants who underwent a periodontal examination were included in the analysis.

### 2.2. Dependent Variables

Highly trained dentists, who are continuously evaluated for quality, performed the periodontal examinations. The community periodontal index (CPI) was determined with reference to the testing criteria proposed by the World Health Organization (WHO) [18]. Periodontal depth was measured at six sites (maxillary right posterior, maxillary anterior, maxillary left posterior, mandibular right posterior, mandibular anterior, and mandibular left posterior) using a WHO probe (3.5 mm and 5.5 mm). The probe tip was gently inserted into the gingival sulcus of the periodontal socket and detected with a force of 20 g. The total buccal and lingual surface of the sulcus of pocket was explored.

The CPI criteria were: 0 (healthy and no signs of inflammation), 1 (gingival bleeding), 2 (calculus), 3 (shallow pocket; >3.5 mm and ≤5.5 mm), and 4 (deep pocket; >5.5 mm). For CPI record, index teeth or teeth remaining in the sextant were evaluated, and the highest score was recorded. In this study, we defined a pocket depth of below 4 mm as normal (CPI 0–2) and ≥4 mm as periodontal diseases (CPI 3, 4).

### 2.3. Independent Variables

#### 2.3.1. Diet Quality

Diet quality was assessed using the Korean Healthy Eating Index (KHEI) [19,20]. The KHEI comprises eight adequacy components (breakfast, whole grains, fruits, vegetables, meat/fish/eggs and beans, and milk and dairy), three moderation components (saturated fatty acid, sodium, sugars), and three balance components (carbohydrates, total fat, and total energy). The KHEI is an index for the overall diet quality of Korean adults. The KHEI was calculated based on the food frequency questionnaire (FFQ) administered by a skilled dietician and the 24-h recall data in the nutritional survey portion of the KHNANES. The total scores range from 0–100, and a higher score indicates a healthier diet. In this study, a KHEI of 80 or higher was defined as “yes,” meaning that the individual has a high-quality diet [14,15].

#### 2.3.2. Physical Activity

Daily physical activities were assessed using the International Physical Activity Questionnaires (IPAQ) long form [21,22]. Physical activity is classified into moderate-intensity activity and high-intensity activity. Moderate-intensity activity causes a mild shortness of breath or mild elevation of heart rate. High-intensity activity is a vigorous activity that causes severe shortness of breath or rapid heart rate.

In this study, adequate physical activity was defined as engaging in 2.5 h or more of moderate-intensity physical activity, 1.25 h of high-intensity physical activity, or a combination of moderate-intensity and high-intensity physical activity (1 min of high-intensity activity = 2 min of moderate-intensity activity) per week. Engaging in adequate physical activity was set as “yes,” and other responses were set as “no” [23].

#### 2.3.3. Normal Body Weight

In this study, normal body weight was determined based on the body mass index (BMI). With reference to the WHO criteria for the Asian population, BMI was classified into five groups: underweight (<18.5 kg/m^2^), normal weight (18.5–22.9 kg/m^2^), overweight (23–24.9 kg/m^2^), obesity (25–30 kg/m^2^), and pathologic obesity (≥30 kg/m^2^) [24]. As previously study, those with BMI < 18.5 were considered to have unhealthy weight and classified as “others” [14].

#### 2.3.4. Healthy Lifestyle Score (HLS Score)

The HLS score was determined based on the presence of the three aforementioned HLS factors. The basic items of the HLS score were selected based on a previous study [14]. The specific criteria of HLS was determined based on the references of each item in Section 2.3.

The score ranged from 0–3, depending on the practice of maintaining diet quality (KHEI ≥ 80; yes), adequate physical activity (yes), and normal weight (18.5–22.9 kg/m^2^; Normal). A higher score indicates higher levels of HLS practices. 

### 2.4. Covariates

The covariates were recategorized as follows, according to the purpose of the analysis. Demographic factors included sex, age, and household income. Age was categorized into 19–29 years, 30–39 years, 40–49 years, 50–59 years, and ≥60 years. Household income was classified as <25% (the lowest quartile group), 25–49%, 50–74%, and 75–100% (the highest quartile group).

Diabetes mellitus was determined based on a fasting glucose level of ≥126 mg/dL, having a physician’s diagnosis, the use of drugs to control blood sugar or insulin injection [25]. Smoking status was classified as non-smokers (<5 packs of cigarettes per lifetime) and current smokers (≥5 packs of cigarettes) [26,27]. Alcohol consumption was assessed using the monthly drinking rate of consuming at least one drink per month in the past year [28]. 

### 2.5. Statistical Analysis

The KNHANES uses a complex sampling design, so we analyzed the data using complex sample methods with weights, strata, and primary sampling units.

The prevalence of periodontal diseases according to general characteristics and HLS score were analyzed using chi-square tests. The associations of each HLS factor (diet quality, physical activity, normal body weight) and total HLS score with periodontal diseases were analyzed using univariate logistic regression and multivariable logistic regression analyses. Model 1 is unadjusted, and Model 2 is adjusted for demographic factors (sex, age, household income). Model 3 is adjusted for Model 2 and for diabetes mellitus, smoking, and drinking. The results were presented as odds ratio (OR) and 95% confidence interval (CI) for periodontal diseases. All statistical analyses were performed using the IBM SPSS Statistics for Windows, Version 26.0 (IBM Corp., Armonk, NY, USA) software. Significance was determined based on a type 1 error of 0.05.

## 3. Results

### 3.1. Prevalence of Periodontal Diseases According to General Characteristics

Table 1 shows the prevalence of periodontal diseases according to participants’ general characteristics. 

The prevalence of periodontal diseases was higher among men (52.1%) than women and increased with advancing age. It was also higher in the middle-low-income group (27.2%) than the high-income group. Among people with periodontal diseases, 15.5% had diabetes mellitus, and 76.2% and 53.2% were non-smokers and alcohol drinkers, respectively. The prevalence of periodontal diseases significantly differed according to all general characteristics (*p* < 0.001).

### 3.2. Prevalence of Periodontal Diseases According to HLS Score

Table 2 shows the prevalence of periodontal diseases according to HLS score. Of all participants, the percentages of participants with a high-quality diet, adequate physical activity, and normal weight were 12.1%, 44.0%, and 38.1%, respectively. The most common HLS score was 1 (45.9%), and the least common HLS score was 3 (2.6%).

Among people with periodontal diseases, the percentages of participants with a high-quality diet, adequate physical activity, and normal weight were 12.2% (*p* = 0.810), 38.1%, and 31.2%, respectively (*p* < 0.001). The most common HLS score was 1 (43.6%).

### 3.3. Association between HLS and Periodontal Diseases

Table 3 shows the ORs for periodontal diseases according to HLS. In Model 3, which adjusted for demographic and health factors, low diet quality, no physical activity, and abnormal weight were associated with periodontal diseases (OR: 1.32, 95% CI: 1.13–1.55, OR: 1.16, 95% CI: 1.04–1.30, OR: 1.26, 95% CI: 1.14–1.40, respectively).

### 3.4. Association between HLS Score and Periodontal Disease

Table 4 shows the associations between HLS and periodontal diseases. In Model 3, which adjusted for all covariates, an HLS score of 0 was significantly associated with periodontal diseases compared to an HLS score of 3 (OR: 1.54, 95% CI: 1.10–2.15).

## 4. Discussion

In this study, we analyzed the raw data of the KNHANES VII, a nationally representative sample of the Korean adult population, to investigate the association between HLS (diet quality, adequate physical activity, normal weight) and periodontal diseases. The key findings on the associations of the total HLS score and each HLS factor with periodontal diseases are discussed here. 

The percentage of participants with a low HLS score was higher in the periodontal diseases group than in the healthy periodontal tissue group (Table 2). However, there was no significant difference in diet quality between the two groups (*p* = 0.810). 

In a model that adjusted for demographic factors and health behavior factors, low diet quality was significantly associated with periodontal diseases (OR: 1.32 95% CI: 1.13–1.55). Various nutrients consumed are used as the components of periodontal tissue. These nutrients also interact with immune cells in the body to play a critical role in immune responses and impact periodontal health [29,30]. 

In the past, studies have generally focused on examining the association between a specific nutrient and oral diseases. However, the use of a quality of eating index is now recommended for a more comprehensive approach to address the multitude of methodological limitations and the multifactorial nature of the disease [31]. A systematic review of health behaviors reported that six studies on diet intake showed substantial improvements of bleeding index and periodontal tissue state following dietary interventions [7]. A healthy diet quality helps reduce severe periodontitis [32]. A systematic review and meta-analysis of diet and chronic diseases reported that a high-quality diet lowers the risk for diabetes mellitus and cardiovascular diseases [33]. These pieces of evidence suggest that a good quality diet will have a positive impact on periodontal diseases. Furthermore, a study on Korean adults found that the diet quality score increased with increasing number of teeth [8]. As shown here, number of teeth affects food choices, dietary changes, imbalanced nutrient intake, and meal quality. Loss of teeth may eventually lead to nutrient deficiency and deteriorated diet quality. Hence, diet quality is anticipated to influence periodontal tissues, which in turn can affect the diet quality.

Physical activity was associated with periodontal diseases (OR: 1.16 95% CI: 1.04–1.30). This result is consistent with previous findings that show that engaging in the recommended amount of physical activity lowers the prevalence of periodontal diseases [34,35]. Regarding the mechanisms through which physical activity affects periodontal tissues, studies have reported a negative correlation between physical activity and plasma inflammatory markers such as C-reactive protein [36,37]. So, physical activity is anticipated to serve as a protective factor for periodontal tissues. In addition, studies have suggested that physical activity prevents type 2 diabetes mellitus—a known risk factor for periodontal diseases—by improving insulin sensitivity [38,39]. Diabetes mellitus can be involved in the elevation of inflammatory markers in the body [40], and hyperglycemia increases the risk and severity of periodontal diseases by inducing inflammation, oxidative stress, and cell apoptosis [41,42]. Thus, preventing diabetes mellitus through physical activity would also positively impact periodontal health.

Abnormal body weight was associated with periodontal diseases (OR: 1.26 95% CI: 1.14–1.40). Previous reports support the association between obesity and periodontal diseases. Obesity has been reported to contribute to the onset of periodontal diseases by elevating TNF-α levels. TNF-α promotes the breakdown of alveolar bones by facilitating osteoclast production and host immune response, thereby accelerating the progression of periodontal diseases [43]. Obesity-related inflammation facilitates bacterial proliferation and releases inflammatory markers in fat tissues and, thus, induces periodontal diseases. Periodontal diseases, in turn, stimulate the production of proinflammatory cytokines that may induce obesity and chronic diseases and impact susceptibility to systemic diseases [44,45].

A systematic review on the association between obesity and infection reported that BMI can influence responses to a particular infection. Moreover, obesity increases exposure to infection and elevates the severity of inflammation and risk of complications [46]. Key systemic conditions such as the metabolic syndrome (abdominal circumference, hypertension, diabetes, and hypercholesterolemia) are associated with masticatory dysfunction, supporting the interrelationship between oral and general health [47]. 

Additionally, it has been reported to contribute to infection by diminishing immune responses, causing chronic inflammation, elevating leptin levels, provoking abnormal blood glucose levels, and stimulating the secretion of inflammatory hormones and cytokines [46,48,49]. In contrast, being underweight is linked to the risk of having a weakened immune system due to nutritional deficiency and inadequate intake of essential amino acids and vitamins. Therefore, being under and overweight can be considered a marker for the potential risk of tooth loss in adults [50].

As shown here, abnormal weight is thought to adversely impact the management and outcomes of periodontal diseases in relation to immune-related factors. An HLS score of 0 was associated with periodontal diseases (OR: 1.54, 95% CI 1.10–2.15). 

A study on three health-enhancing behaviors using the NHANES III data reported that the OR for periodontal diseases decreased with increasing number of health-enhancing behaviors [14]. In our study, however, the risk for periodontal diseases did not significantly decline with increasing HLS score. Nevertheless, an HLS score of 0—not engaging in any of the three HLS practices—or poor HLS practices was associated with periodontal diseases.

Dental professionals should provide interventions and support to promote health behaviors in patients to improve their diet quality, physical activities, and body weight. The effectiveness of such interventions has been substantiated in previous studies, thereby confirming the need for counseling interventions to promote health behaviors [7]. Therefore, in addition to improving oral hygiene—the primary focus in the past—improving HLS should be emphasized for patients with periodontal diseases. Moreover, comprehensive approaches through interprofessional collaboration should be taken depending on the results of patients’ assessments.

This study has the following limitations. First, the CPI test used for classification of periodontal disease did not evaluate the loss of attachment, which can estimate the lifetime cumulative failure rate of periodontal attachment. Therefore, further studies should apply the study using the newly updated 2018 World Workshop periodontitis definition [51]. Second, the BMI and HLS score used in this study did not include alternative measures, which may have led to over- or underestimation of the results. Third, the criteria for diabetes mellitus applied in this study were determined based on fasting glucose level, physician’s diagnosis, and the use of drugs. Hence, HbA1c, used for the diagnosis and follow-up of diabetes mellitus, was not considered. Therefore, there are limitations in distinguishing between under- and over-influence on diabetes mellitus and between type 1 and type 2 diabetes mellitus. Fourth, in contrast to previous studies, this study was conducted exclusively on the Korean adult population, and findings can differ across races and biological features. Additionally, the causality among the study parameters cannot be established due to the cross-sectional nature of this study. Notwithstanding these limitations, this study has several strengths. This study is one of the first studies to investigate the associations of the overall HLS score and each of the three HLS factors with periodontal diseases in the Korean adult population. Furthermore, poor practice of HLS was identified as a risk factor for periodontal diseases. Thus, subsequent studies should investigate the association between HLS and periodontal diseases in patients with chronic conditions that are strongly associated with periodontal health, such as diabetes mellitus. Additionally, we recommend that researchers conduct longitudinal studies to analyze the trends of changes in periodontal health in relation to the degree of HLS practice.

## 5. Conclusions

Each of the three factors of HLS (diet quality, adequate physical activity, normal body weight) was associated with periodontal diseases. In particular, poor practice of HLS was related to periodontal disease. Hence, individuals should manage their health by ensuring a high-quality diet, engaging in adequate physical activities, and maintaining a normal body weight, and further studies are required.

## Figures and Tables

**Table 1:** Characteristics of the study population stratified by periodontal diseases.

Characteristics		Periodontal Disease				*p*-Value **
		No		Yes		
		*n*	% *	*n*	% *	
Sex (*n* = 12,689)	Man	3417	37.8	2128	52.1	<0.001
	Woman	5291	62.2	1853	47.9	
Age (*n* = 12,689)	19–29	1501	17.8	61	1.7	<0.001
	30–39	1806	20.0	294	7.1	
	40–49	1768	19.8	647	15.2	
	50–59	1466	17.8	1005	26.4	
	≥60	2167	24.5	1974	49.6	
House income (*n* = 12,656)	Low	1331	15.1	1009	25.0	<0.001
	Middle low	2011	22.8	1077	27.2	
	Middle high	2552	29.1	992	25.1	
	High	2796	33.0	888	22.7	
Diabetes (*n* = 12,689)	Absence	8134	93.7	3358	84.5	<0.001
	Presence	574	6.3	623	15.5	
Smoking (*n* = 12,571)	Non-smoker	7344	85.4	2966	76.2	<0.001
	Smoker	1292	14.6	969	23.8	
Alcohol drinking (*n* = 12,580)	Non-drinker	3921	49.8	520	46.8	<0.001
	Drinker	4716	50.2	2113	53.2	

* Weighted %; ** *p*-Value was calculated by complex sample chi-square test.

**Table 2:** Prevalence of Periodontal Diseases According to HLS Score.

HLS	*n*	%	Periodontal Diseases Prevalence				*p*-Value **
			No		Yes		
			*n*	% *	*n*	% *	
Diet quality							
No	10,132	87.9	6643	87.6	3057	87.8	0.810
Yes	1357	12.1	915	12.4	410	12.2	
Physical activity							
No	7196	56.0	4500	52.5	2352	61.9	<0.001
Yes	5357	44.0	3832	47.5	1411	38.1	
Normal body weight							
Others	8278	61.9	5185	58.9	2771	68.8	<0.001
Normal	4921	38.1	3523	41.1	1210	31.2	
HLS score							
0	3599	31.3	2091	27.3	1305	38.5	<0.001
1	4992	45.9	3410	47.3	1424	43.6	
2	2129	20.2	1579	22.6	491	15.5	
3	269	2.6	192	2.9	76	2.4	

* Weighted %; ** *p*-Value was calculated by complex sample chi-square test; HLS score = Healthy lifestyle score.

**Table 3:** Multivariable association between HLS and periodontal diseases.

HLS	Model 1	Model 2	Model 3
	Unadjusted	Adjusted Odds Ratio (95% Confidence Interval)	
Low diet quality (Ref. Yes)	1.02 (0.89–1.17)	1.33 (1.15–1.55) ***	1.32 (1.13–1.55) **
Insufficient physical activity (Ref. Yes)	1.47 (1.34–1.62) ***	1.19 (1.07–1.32) **	1.16 (1.04–1.30) *
Abnormal body weight (Ref. Normal)	1.54 (1.40–1.69) ***	1.24 (1.12–1.37) ***	1.26 (1.14–1.40) ***

Data are presented as OR (95% CI). OR: odds ratio; CI: confidence interval, * *p* < 0.05, ** *p* < 0.01 and *** *p* < 0.001. Model 1 unadjusted model. Model 2 adjusted for demographic factors (sex, age, and household income). Model 3 adjusted for the same factors as model 2 plus health factors (diabetes mellitus, smoking, and alcohol drinking); HLS = Healthy lifestyle.

**Table 4:** Multivariable association between HLS score and periodontal diseases.

	Model 1	Model 2	Model 3
	Unadjusted	Adjusted Odds Ratio (95% Confidence Interval)	
HLS score 0	1.67 (1.22–2.29) **	1.58 (1.13–2.20) **	1.54 (1.10–2.15) *
HLS score 1	1.09 (0.81–1.48)	1.27 (0.93–1.75)	1.24 (0.90–1.72)
HLS score 2	0.81 (0.59–1.12)	1.07 (0.76–1.50)	1.05 (0.74–1.49)
HLS score 3	Reference	Reference	Reference

Data are presented as OR (95% CI). OR: odds ratio; CI: confidence interval, * *p* < 0.05, ** *p* < 0.01. Model 1 unadjusted model. Model 2 adjusted for demographic factors (sex, age, and household income). Model 3 adjusted for the same factors as model 2 plus health factors (diabetes mellitus, smoking, and alcohol drinking); HLS score = Healthy lifestyle score.

## Data Availability

The data from the KNHANES VII survey can be accessed and downloaded from the KNHANES homepage (URL: https://knhanes.kdca.go.kr/knhanes/eng/index.do accessed on 7 February 2022).
